# Basophil Activation as Marker of Clinically Relevant Allergy and Therapy Outcome

**DOI:** 10.3389/fimmu.2020.01815

**Published:** 2020-08-21

**Authors:** Bernadette Eberlein

**Affiliations:** Department of Dermatology and Allergy Biederstein, School of Medicine, Technische Universität München, Munich, Germany

**Keywords:** basophil activation test, basophil parameters, food allergy, hymenoptera venom allergy, inhalant allergy, drug allergy, immunotherapy, anti-IgE-treatment

## Abstract

For some years now the basophil activation test (BAT) using flow cytometry has emerged as a powerful tool and sensitive marker that can be used to detect clinically relevant allergies, provide information on the severity of an allergic reaction, and monitor therapies. Compared to other *in vitro* diagnostic tests, BAT seems to have a better informative value in terms of clinical relevance. In general, the BAT can be used for the diagnosis of the most common forms of IgE-mediated allergy such as hymenoptera venom allergy, inhalant allergy, food allergy, and drug allergy. Various basophil markers and parameters have been established which, depending on the trigger of the respective allergy, can provide information on the clinical relevance of sensitization, on the development of natural tolerance, on trigger thresholds, and on the severity of the allergic reaction. The BAT also serves as a suitable follow-up instrument for various therapeutic approaches such as specific immunotherapy, desensitization protocols, or use of anti-IgE-antibodies for the various diseases. Quality controls for routine use, standardization, and automatization are expected to expand the range of applications for the above-mentioned indications.

## Introduction

Cellular *in vitro* tests can be used for the allergy diagnosis of type I allergies and serve for the detection of indirect sensitization on basophils (due to their easier availability compared to mast cells). In recent years the basophil activation test (BAT) which measures activation markers after incubation with allergens or other triggers by flowcytometry has emerged as the most widely used test for this purpose.

In most studies the activation marker CD63 was favored, occasionally also CD203c. CD63, a membrane component of the basophil granules, is not a basophil-specific marker and is also expressed on other blood cells. Therefore, further labeling is necessary for the identification of basophils. Possible markers include anti-CCR3, anti-IgE, anti-CRTH2, CD203c, or anti-CD123. CD203c, an ectoenzyme located both on the plasma membrane and in the cytoplasmic compartment of basophils, is a basophil-specific marker and is expressed constitutively. The test can be performed with full blood, washed basophils, or donor basophils. This and various protocols are the main differences between the BATs used in different laboratories. CD203c and CD63 markers are upregulated after IgE receptor aggregation but have partially different metabolic pathways and follow different kinetics. Interleukin-3 potentiates the allergen-induced CD63 expression without upregulating CD63 itself, whereas it increases CD203c expression even without allergen.

Results of the BATs are usually expressed as percentages of activated basophils (% CD63+ cells), sometimes also as MFI (mean fluorescent intensity). This basophil reactivity measures the number of basophils that respond to a given stimulus. Maximal basophils reactivity is the maximal activity induced by a given stimulus. Additionally, further parameters such as results of the determination of the half-maximal concentration (EC50, CD-sens, basophil sensitivity), the calculation of a ratio (CD63 ratio), of allergen-induced CD63 activation in comparison to an IgE-dependent positive control (anti-IgE of anti-FcεRI), or of the area under the curve (AUC) in dose-response curves turned out to be of value for the assessment of clinically relevant allergies and therapy outcomes (1–4). Details can be found in an EAACI position paper (1).

## Elucidation of Clinically Relevant Allergy

### Food Allergy

For food allergies, the sensitivity of the BAT varies between 62 and 90% and the specificity between 80 and 100% depending on the allergen. In general, cellular tests are useful to detect the trigger of an IgE-mediated reaction to food if conventional diagnostics is negative or not available and a provocation test is expected to be potentially life-threatening. In recent years, more and more studies have been published which see the basophil activation test as a diagnostic tool prior to oral provocation being only necessary in remaining unclear cases (1).

In 2014, Santos et al. could show that the BAT discriminates between allergy and tolerance in peanut-sensitized children. Receiver operator curves (ROC) showed that the BAT with a peanut extract was better than skin prick test (SPT) and sIgE to Ara h 2 and peanut for this purpose. The application of BAT as a second or third step in the diagnostic workup dramatically reduced the need for oral provocation tests. It was recommended to perform oral food challenges in cases with equivocal BAT as well as in BAT-negative patients (5). Other authors showed that a negative CD-sens to peanut of Ara h 2 excluded an allergy (6). Certain parameters of the BAT using a peanut extract correlated with the severity of the reaction (CD63 ratio) and with the amount of eliciting allergen (CD-sens) (2, 7). Interestingly, only the use of a peanut extract and not of Ara h 2 in the BAT was associated to the eliciting dose of peanut in allergic patients (8).

In milk allergic children BAT helped in deciding when to reintroduce cow's milk in their diet showing that CD63 ratio reflected the severity of reaction to oral challenge (9). This parameter was also significantly higher among patients with milk allergy who reacted to baked milk than among those who tolerated it (10). As a consequence, the BAT reduced the need for a food challenge in children suspected of IgE-mediated cow's milk allergy (11).

Baked egg-reactive children had significantly increased basophil activation in response to intermediated stimulation levels of egg white protein compared to tolerant children, but there was a great overlap in basophil activation between these groups, which made it difficult to use it in clinical practice (12).

CD63 and CD203c expression at several allergen concentrations differed between individuals allergic or sensitized to hazelnut, too. In this study, EC50 of allergen-induced CD203c expression displayed a better discrimination compared to CD63, but there was no significant difference between patients with oral allergy syndrome and systemic reactions (13).

Similarly, basophil activation with peach extract was higher in mugwort pollen-related peach allergic patients than in tolerant subjects, but the BAT results were comparable in patients with oral allergy syndrome and systemic reactions, limiting its utility in predicting severity. In contrast, the basophil activation with Pru p 3 correlated not only with clinical allergy but also with the severity of symptoms having the best diagnostic performance compared to determination of sIgE (14).

Also for rare food allergies, e.g., the alpha-gal syndrome, it could be shown that the BAT differentiates between patients with a clinically relevant allergy and asymptomatic alpha-gal sensitization. Especially the parameter CD63 ratio for low concentrations of alpha-gal turned out to be a reliable basophil parameter and was better than sIgE to alpha-gal (4).

In another study it was shown that the BAT using hydrolyzed wheat protein and ω-5 gliadin was highly useful for diagnosing the subtypes of hydrolyzed wheat protein WDEIA (wheat-dependent exercise-induced anaphylaxis) and conventional WDEIA indicating an IgE-response to different protein components (15). Despite a tendency to higher wheat CD-sens values, only the combination of CD-sens and sIgE to wheat or wheat components was useful in the prediction of wheat challenge outcome (16).

Due to good results of CD203c sesame-induced basophil expression joint utilization of BAT and skin prick test with a high protein concentration sesame extract, this approach may also obviate the need for oral food challenge in most patients with sesame food allergy (17).

### Hymenoptera Venom Allergy

For hymenoptera venom allergies, the sensitivity for the BAT varies between 85 and 100% and the specificity between 83 to 100% (1). There is no correlation between basophil activation and the clinical severity of the sting reaction reported by patients (18).

Because diagnostic sting challenges for insect venom allergies are not performed routinely for ethical reasons, this cellular test can be used in diagnostics for the detection of an IgE mediated reaction, especially if skin tests and specific IgE antibodies to insect venom extracts are negative (19). Although the component resolved diagnosis has made significant progress in specific IgE determination for insect venom allergic patients, there are still individuals in which only the BAT showed positive results (20). The use of recombinantly produced CCD-free hymenoptera venom allergens also lead to an improvement of the BAT results compared to the total hymenoptera venom extracts, both in terms of the number of positive results and the level of activation (21).

The BAT turned out to be helpful also in cases of double sensitization to bee and vespid venom and a clinical reaction to only one insect species or in cases of insect stings that cannot be clearly assigned to a particular insect species from the clinical history. In about one third of the patients information about the clinically relevant insect could be obtained by the BAT incubating the cells with bee and wasp venom extracts and, if necessary, by calculating the half-maximum concentration of the dose-response curves and forming a ratio (22–24). The clinical relevance of such BAT results could be confirmed in patients with double sensitization (skin test and specific IgE antibodies) and exclusive monosensitization to vespid venom in the BAT: 92% of the patients tolerated a sting challenge test with the bee (BAT negative) without systemic reaction, and 7% suffered from a mild systemic reaction (25). Thus, unnecessary specific immunotherapy can be avoided.

### Inhalant Allergy

The sensitivity of the BAT for house dust mites, pollen, latex, or cat hair is 91–100% for both extracts and recombinant major allergens, and the specificity is between 96 and 100% (1).

Due to the good sensitivity of conventional diagnostics, cellular tests are used less for diagnostic purposes in routine, but the usefulness of the BAT and component-resolved diagnosis in distinguishing between symptomatic allergic rhinitis patients and asymptomatic sensitization to house dust mite could be demonstrated. Symptomatic patients showed a lower threshold for *in vitro* basophil activation and a higher AUC. There was also a positive correlation between the number of recognized house dust mite allergens and the AUC of basophil activation (26).

BAT seems to be advantageous in the diagnosis of local allergic rhinitis (LAR) because it was able to diagnose at least 50% of these cases allergic to house dust mite extracts and was more sensitive than detection of nasal specific IgE and less time-consuming than nasal provocation tests (27, 28). Similar results were shown for LAR patients with olive tree pollen (29). Based on these studies BAT has been shown to have a sensitivity of 50.0–66.6% and a specificity of 90.0–91.7% in LAR. These results reinforce the usefulness of BAT, a rational step of a diagnostic approach in LAR before nasal provocation tests.

### Drug Allergy

In general, sensitivity of the BAT for most drugs is significantly lower than the sensitivity of the allergens mentioned above. The sensitivity of the BAT for beta-lactam antibiotics is about 50% with a positive predictive value of about 90%. In order to obtain relevant information about the sensitization of a patient by this test, the BAT should be carried out within half a year after the clinical reaction, since the cells' reactivity to the antibiotics decreases thereafter. Sensitivity for quinolones is slightly better (about 64%) with a positive predictive value of about 90% (30).

The sensitivity of BAT in hypersensitivity reactions to NSAIDs being independent of IgE-/FcεRI cross-linking is very low (20–40%) with specificities of 40–100%; only BAT with pyrazolones showed better results (sensitivity about 54%, specificity about 95%) (30, 31).

For radio contrast media the sensitivity is about 60% with positive predictive values of about 97%. The sensitivity for muscle relaxants varies between 54 and 92% for BAT (specificity: 100%) with a positive predictive value of about 96% (30). Algorithms for allergy workup in perioperative hypersensitivity reactions include the BAT before considering drug provocation tests: Negative skin testing and BAT results might increase confidence in performing drug provocation tests (32–34).

The studies to date show that cellular tests with drugs should only be used as a supplement to existing diagnostics, and they are not a substitute for provocation tests (30).

## Therapy Outcome

Over the last few years, it has become apparent that the BAT can serve as a suitable follow-up instrument for various therapeutic approaches such as specific immunotherapy, desensitization protocols, or use of anti-IgE-antibodies for various allergic diseases.

### Immunotherapy in Food Allergy

During a 12-months sublingual immunotherapy (SLIT) for peanut allergy in children a significantly decreased basophil activity after stimulation with the two lowest concentrations of a crude peanut extract could be demonstrated (35). Others showed that 2-years responders of a SLIT had significantly lower percent CD63+ basophils than non-responders for the lower peanut stimulant levels, but there are also studies demonstrating that peanut-induced basophil response was most reduced in the immune tolerant group after 24 months of oral immunotherapy (OIT), although differences between immune tolerant and non-tolerant participants did not achieve statistical significance (36, 37). Using the CD63 ratio with a crude peanut extract, a significant decrease of this parameter at all concentrations after 3 to 5 years of peanut SLIT was observed (38).

In a pilot study the utility of BAT for monitoring the acquisition of clinical tolerance after oral desensitization to cow's milk over 12 months was shown (39). Furthermore, milk-induced %CD63 and %CD203c expression was significantly lower in patients >24 months of oral immunotherapy vs. in patients <24 months of treatment (40).

Also, a decrease in antigen-specific CD63 basophil expression (egg white, ovomucoid, ovalbumin) was associated with the development of tolerance to egg by specific oral tolerance induction after 15 days and 1 month, respectively, of the buildup phase (41, 42).

In contrast, a 6 month or 12 month SLIT with a peach extract lead to an increase in basophil activation following stimulation with rPru p3 (43, 44).

### Immunotherapy With Hymenoptera Venoms

A basophil activation decrease using mostly submaximal concentrations of insect venoms was only observed in part of the studies up to 18 months after beginning of venom immunotherapy (VIT), but was found throughout all studies after 2 years of treatment, and maintained until the completion of a 3–5-years immunotherapy period (45–50). A significant difference was also shown for submaximal concentrations of bee venom in patients reacting to a sting challenge compared to patients not reacting at the end (mean 4.4. years) of VIT (51). The depression of allergen-specific basophil response also lasted 1 year after completing 4–6.5 years of immunotherapy (47). In a BAT inhibition assay incubating blood of donor patients with insect venom allergy with sera from patients undergoing VIT for at least 1 year, the basophil response was almost completely inhibited at submaximal allergen concentrations (52). It was shown that patients who reacted after discontinuation of immunotherapy in field re-stings had a persistence of high basophil activation at submaximal concentrations in contrast to protected patients (53).

### Immunotherpy With Inhalant Allergens

First indications of the benefit of BAT for the monitoring of specific immunotherapy (SIT) with pollen were shown in patients with Japanese cedar pollinosis. Significant reductions in the allergen-induced CD203c response in basophils were observed in part of the subjects already 1 month after beginning of a rush immunotherapy (54). CD-sens dropped significantly after reaching the maintenance dose of SIT for birch or grass allergy compared to before (55). Similarly, a decrease in allergen-induced basophil activation at submaximal allergen concentrations was demonstrated at the end of a short-term preseasonal immunotherapy over 7 weeks and additionally at the peak of the grass pollen season after immunotherapy (56). CD63 expression decreased also 8 months after an immunotherapy with an olive pollen allergoid compared to baseline values (57). Basophil sensitivity was significantly lower after 1 month of treatment with subcutaneous immunotherapy (SCIT) to grass pollen when compared to SLIT-tablet treatment, and although the differences diminished towards the end of the study (15 months), they remained significant (58). Interestingly, a decrease in basophil sensitivity after 3 weeks of treatment predicted long-term improvement in seasonal combined symptom and medication scores during 3 years of treatment in grass pollen allergic patients (59). Grass pollen immunotherapy induced sustained suppression of the allergen-specific basophil response during initiation and after 1–2 years after completion of treatment (60). In contrast to these studies, a significant decrease in the basophil activation to various grass allergens was not found after 2 or 4 months of a SLIT with a five-grass-pollen tablet vs. placebo using a defined allergen challenge chamber (61).

For house dust mite (HDM) allergy a significant decrease in BAT results in the course of specific immunotherapy with HDM allergens in children was shown. CD-sens seemed to be a better monitoring parameter than the plain percentage of CD63-expressing basophils (62). Another study demonstrated that after the first and second year of HDM immunotherapy, CD63 expression was lower in atopic dermatitis active group than in the atopic dermatitis control group (63), but others did not find a significant change of basophil reactivity to HDM during 24 months of immunotherapy nor a significant association between the change in clinical symptoms and a change in basophil reactivity (64). A phase I study with timothy grass and dust mite dual-SLIT for pollen allergy showed that basophil activation for these two allergens decreased after 24 months of SLIT compared to baseline (65).

During a latex sublingual immunotherapy in children BAT determinations showed significant decreases in recombinant and natural latex allergens in the active group at 6 months, but not at 12 months (66).

### Desensitization of Drugs

It was shown in single cases that desensitization protocols can be monitored by the decrease of basophil sensitivity to the eliciting drug. This was published for insulin, pertuzumab, adalimumab, and brentuximab (67–70). For other drugs, e.g., etanercept and platinum compounds, this could not be constantly demonstrated (71, 72).

### Anti-IgE Treatment

In patients with chronic urticaria in whom omalizumab is licensed there was no significant difference in activation of donor basophils incubated with patients' serum before and after 3 months of treatment (73).

In contrast, in patients with peanut allergy, individually dosed omalizumab *in vivo* could be monitored by CD-sens based on peanut induced basophil activation *in vitro* and facilitated peanut oral immunotherapy (74, 75). In severe cow's milk allergy, CD-sens monitoring during omalizumab treatment helped in the decision for performing food challenge (76).

Timothy allergic patients who received omalizumab for 3 months had a decline in CD-sens during the treatment and stayed below the starting value for at least 3 months after the treatment (77). A decrease of CD-sens after a 4-months treatment with omalizumab was also seen in cat allergic patients (78). Furthermore, 12–14 months after closing of 6-years omalizumab treatment, a downregulation of basophil reactivity was still seen (79).

## Conclusion and Perspectives

This overview showed that the flowcytometric measurement of allergen-induced basophil activation and the calculation of basophil parameters from the dose-response curves could help to gain better estimates of *in vivo* reactions in a number, but not all type-I allergic diseases in comparison to conventional diagnostics (Table 1). Especially the consideration of results in the submaximal allergen range proved to be particularly relevant and should be pursued further. A thorough characterization of the patients which were not completely transparent in all studies is a prerequisite. Furthermore, quality controls for routine use, standardization, and automatization are expected to expand the range of applications for the above-mentioned indications.

**Table 1:** Overview over possible current applications of BAT for discrimination between clinically relevant allergy and tolerance, monitoring immunotherapy, and follow-up of anti-IgE treatment for food, hymenoptera, and inhalant allergies according to the literature.

**Allergy**	**Allergen**	**Discrimination between allergy and tolerance/sensitization**	**Monitoring immunotherapy (IT)**	**Follow-up of anti-IgE treatment**	**Comments**	**References**
Food						
	Peanut	Yes	Yes (1–5 years IT)	Yes		(5, 6, 35–38, 74, 75)
	Milk (baked)	Yes	Yes (1–2 years IT)	Yes		(9–11, 39, 40, 76)
	Egg, baked	Partially^a^	Yes (15 days to 1 month IT)		^a^Great overlap between groups	(12, 41, 42)
	Hazelnut	Yes^b^			^b^No discrimination between OAS and systemic reaction	(13)
	Peach	Yes^c^	No		^c^No discrimination between OAS and systemic reaction	(14, 43, 44)
	Alpha-Gal	Yes				(4)
	Sesame	Yes (together with SPT)				(17)
	Wheat	Yes (together with sIgE)			Discrimination between subtypes of WDEIA possible	(15, 16)
Hymenoptera						
	Bee and wasp venom	Yes (in terms of IgE-mediated reaction and of double sensitization)	Yes (1.5–5 years IT and > 1 years after the end of IT)			(20–25, 45–53)
Inhalant						
	Pollen (Japanese cedar, grass, olive pollen allergoid)	Yes^d^	Yes, in most studies^e, f^	Yes	^d^Especially for LAR ^e^SCIT better than SLIT ^f^Not for a five-grass pollen tablet	(27–29, 54–61, 77)
	House dust mite	Yes	Yes, in most studies			(26, 62–64)
	Cat			Yes		(78)
	Latex		Yes			(66)

*IT, immunotherapy; LAR, local allergic rhinitis; OAS, oral allergy syndrome; SCIT, subcutaneous immunotherapy; SLIT, sublingual immunotherapy; WDEIA, wheat dependent exercise induced anaphylaxis*.

## Author Contributions

The author confirms being the sole contributor of this work and has approved it for publication.

## Conflict of Interest

BE received methodological and technical support from the company BÜHLMANN Laboratories (Schönenbuch, Switzerland).
